# Antioxidant Therapy Reverses Hepatotoxicity Induced by Microcystin-LR in a Cellular Model of Metabolic Dysfunction-Associated Steatotic Liver Disease (MASLD)

**DOI:** 10.3390/jox16030076

**Published:** 2026-04-29

**Authors:** Apurva Lad, Jason Kindle, Prajwal Hegde, Gabriel G. Kleer, Andrew L. Kleinhenz, Johnna A. Birbeck, Judy Westrick, Nicholas J. Peraino, Terry D. Hinds, Neeraja Purandare, Andrew M. Fribley, Steven T. Haller, David J. Kennedy

**Affiliations:** 1 Department of Medicine, University of Toledo, Toledo, OH 43614, USAprajwal.hegde@vumc.org (P.H.); gabriel.kleer@rockets.utoledo.edu (G.G.K.); andrew.kleinhenz@utoledo.edu (A.L.K.); neeraja.purandare@utoledo.edu (N.P.); andrew.fribley@utoledo.edu (A.M.F.); 2 Department of Chemistry, Wayne State University, Detroit, MI 43614, USAjudy.westrick@wayne.edu (J.W.); nperaino@chem.wayne.edu (N.J.P.); 3 Drug & Disease Discovery D3 Research Center, Department of Pharmacology and Nutritional Sciences, University of Kentucky College of Medicine, Lexington, KY 40508, USA; terry.hinds@uky.edu

**Keywords:** MASLD, oxidative stress, N-acetylcysteine, pNaKtide, obesity, antioxidants, cyanotoxins, microcystin-LR

## Abstract

Microcystin-LR (MC-LR) is a potent hepatotoxin that has been shown to cause liver damage even at doses lower than the established Low Observable Adverse Effect Level (LOAEL) of 200 μg/kg in animal models. We have previously observed that low-dose exposure to MC-LR in animals with diet-induced Metabolic Dysfunction-Associated Steatotic Liver Disease (MASLD) and subsequent treatment with antioxidants like N-acetylcysteine (NAC) and the Na^+^/K^+^ ATPase-Src kinase inhibitor pNaKtide significantly alleviated hepatic infiltration of immune cells, downregulated markers of inflammation and hepatotoxicity, increased the breakdown of the toxin molecule, and restored phase I and II drug metabolism pathways, including the glutathione pathway. Because the liver is composed of heterogeneous cell types, this study aimed to determine the specific role of hepatocytes in the uptake and metabolism of MC-LR, especially in the setting of MASLD. To address this, we used two well-established hepatocyte cell lines—AML-12 murine hepatocytes and human Hep3B hepatocytes. Preliminary dose comparison studies with AML-12 cells showed that MC-LR at 10 μM concentration showed a significant upregulation in the genetic expression of the markers of hepatotoxicity—*OSMR* (*p* ≤ 0.01) and *SerpinE* (*p* ≤ 0.0001)—in comparison to Vehicle. Treatment with pNaKtide (1 µM) and/or NAC (10 mM) in the presence of MC-LR significantly reduced the expression of both *OSMR* (*p* ≤ 0.0001) and *SerpinE* (*p* ≤ 0.01 and *p* ≤ 0.0001, respectively). To model steatotic hepatocytes characteristic of the MASLD phenotype, Hep3B hepatocytes were first treated with 500 µM of oleic acid (OA) before exposing them to the toxin in the presence and absence of antioxidants. MC-LR exposure, induced markers of inflammation and hepatotoxicity to be elevated significantly in the presence of OA as compared to MC-LR exposure alone. This elevation of the genetic markers of inflammation and hepatotoxicity was significantly attenuated on treatment with pNaKtide (1 µM) and NAC (10 mM). Quantification of human SERPINE1 (PAI1) and 8-OHdG, a stable marker of oxidative stress, in the spent media of Hep3B cells corroborated the trends observed in the genetic markers of hepatotoxicity. These observations support the central role that hepatocytes play in the uptake and metabolism of MC-LR, which is complicated by the presence of MASLD-like conditions and can help in the development of future therapeutic strategies.

## 1. Introduction

Cyanobacteria, also called the blue-green algae due to the color of their accessory pigment phycocyanin, are photosynthetic bacteria and are an important aspect of the aquatic food web [1]. Factors such as excess eutrophication of water reservoirs and changing environmental conditions (e.g., warmer temperatures, pH changes, and rising CO_2_ levels) can cause these organisms to form dense blooms [1,2]. It has been noted that the occurrence of these cyanobacterial blooms has been on the rise globally in recent years due to increasing anthropogenic activities, and threatens our ecosystem [3]. Eutrophic blooms negatively impact water quality by increasing turbidity, generating unpleasant odors and tastes, and producing toxic metabolites. These blooms deplete dissolved oxygen in the water, leading to hypoxic conditions that can be lethal to aquatic organisms. Consequently, this disruption can cascade through the aquatic food web, ultimately affecting the entire ecosystem [4]. Some of the common bloom-forming cyanobacterial species found in Lake Erie include *Anabaena*, *Cylindrospermopsis*, *Microcystis*, *Oscillatoria*, and *Planktothrix* spp. [5]. The levels of toxins in the notorious 2014 algal bloom, dominated by the *Microcystis* spp., in the Western basin of Lake Erie were reported to exceed 8 µg/L [6].

Among the various metabolites produced by cyanobacteria, microcystins (MCs) are the most common and are distributed worldwide [7]. There are over 300 different congeners of microcystin, but the most common and potent of these congeners is Microcystin-LR (MC-LR). It is a cyclic heptapeptide characterized by Leucine (L) and Arginine (R) as the variable amino acids in positions 2 and 4, and a unique 3-amino-9-methoxy-2,6,8-trimethyl-10-phenyldeca-4,6-dienoic acid (ADDA) group in position 5 [8,9]. Humans and animals can be exposed to these toxins in three ways—ingestion/oral route, inhalation, or skin contact [10]. Once exposed, it is actively transported inside the cells via the organic anion-transporting polypeptides (OATPs), including *OATP1B1* and *OATP1B3* [11,12]. Once inside the cell, MCs actively target and inhibit members of the protein phosphatase family, particularly protein phosphatases 1 (PP1) and 2A (PP2A), by dephosphorylating the serine and threonine residues via covalent bonding [13]. This cascades into a variety of downstream processes, such as cytoskeletal disruption [14], an increase in oxidative stress and formation of reactive oxygen species (ROS) [15], DNA damage [16], apoptosis [11], endoplasmic reticulum dysfunction [17], and inflammation through activation of the MAPK and NFκB signaling pathways [18]. The liver is a major target organ for microcystins, as demonstrated by multiple studies examining the in vivo biological distribution of the toxin following different routes of exposure [19,20], followed closely by the kidneys [21]. A review by Arman and Clarke summarizes the toxicokinetics of microcystins, their molecular toxicology, and pathophysiology in detail across preclinical rodent models and humans [22].

According to the World Health Organization (WHO) guidelines (2020), a provisional guideline value of 1 µg/L has been established for microcystins (as MC-LR equivalents) in drinking water for lifetime exposure, while higher short-term health-based values (e.g., 12 µg/L for adults over periods up to two weeks) have been proposed for intermittent exposures, reflecting differing risk considerations rather than a defined threshold of safety [23]. These guidelines were established based in part on a 13-week study performed by Fawell et al. in a healthy murine model [24]. However, very little is known about the susceptibility to these toxins in the presence of pre-existing conditions such as Metabolic Dysfunction-Associated Steatotic Liver Disease (MASLD).

MASLD, formerly known as Non-Alcoholic Fatty Liver Disease (NAFLD), is the most common condition of chronic liver disease in Western countries and affects ≥30% of the population [25,26]. The global prevalence of MASLD has increased concurrently with obesity and diabetes by 50% in the last two decades [27]. It is an umbrella term for a diverse range of liver conditions affecting people who drink little to no alcohol. The condition is defined by the presence of steatosis, where greater than 5% of the hepatocytes accumulate lipids [28]. It can progress to MASH (Metabolic Dysfunction-Associated Steatohepatitis) [29], a more severe form of MASLD that is accompanied by fibrosis and inflammation [30]. In rare events, MASLD may progress to hepatocellular carcinoma (HCC). In addition to the liver-related complications, MASLD is also associated with cardiovascular disease (CVD), chronic kidney disease (CKD), and certain types of extrahepatic cancers [27]. It has been noted that patients with MASLD are often associated with other metabolic comorbidities such as impaired insulin response, type 2 diabetes, dyslipidemia, hypertriglyceridemia, and hypertension [31]. MASLD results from metabolic dysregulation, particularly insulin resistance, in which increased fatty acid influx and synthesis exceed the liver’s capacity to oxidize and export lipids, leading to triglyceride accumulation [32]. These factors act as the first hit in reducing a healthy response.

In 2015, a study published by Zhang et al. showed a significant association between the occurrence of cyanobacterial blooms and the prevalence of MASLD in the population of the United States using a novel satellite imaging technique [33]. Thereafter, a 2017 study by He et al. demonstrated that prolonged low-dose exposure to MC-LR in mice markedly altered hepatic proteins involved in lipid metabolism. Their integrated data from proteomic, metabolic, histological, and cytokine profiles revealed that MC-LR significantly inhibited hepatic fatty acid β-oxidation and lipoprotein secretion thereby promoting hepatic inflammation, resulting in nonalcoholic steatohepatitis disease (NASH) or Metabolic Dysfunction-Associated Steatohepatitis (MASH) like conditions. Further studies conducted by Arman and Clarke in a diet-induced MASLD model of rats showed increased hepatic inflammation and fibrosis, plasma cholesterol levels, decreased PP1 and PP2A protein levels, and increased urinary KIM-1 levels on exposure to semi-chronic levels of MC-LR [34,35,36].

Previously, we have shown that chronic low-dose exposure to MC-LR in a genetically modified murine model of MASLD significantly exacerbated hepatic injury, accompanied by genetic and phosphoproteomic dysregulation of key signaling pathways [37]. In a separate study using a diet-induced model of MASLD, we found that hepatic injury exacerbated by exposure to MC-LR was markedly reduced with antioxidant treatment such as NAC and the Na^+^/K^+^ ATPase-Src kinase inhibitor pNaKtide, both of which are known to augment the glutathione detoxification pathway [38]. Common histological observations in MASLD liver sections from mice exposed to MC-LR included ballooning hepatocytes with micro- and macrovesicular lipid accumulation, immune cell infiltration, and lobular inflammation. However, we wanted to validate the role played by hepatocytes alone in toxin metabolism and breakdown. Therefore, this study aimed to better understand the role of hepatocytes in the uptake and metabolism of the cyanotoxin MC-LR using well established cell models.

## 2. Materials and Methods

### 2.1. Cell Lines

#### 2.1.1. AML-12 Hepatocytes

The AML-12 (alpha mouse liver 12) cell line was obtained from American Type Culture Collection (ATCC) (Cat. No. CRL-2254, ATCC, Manassas, VA, USA). The cells were grown in DMEM/F12 1:1 media (Cat. No. SH30261.01, HyClone Laboratories, Logan, UT, USA) supplemented with 10% Fetal Bovine Serum (FBS) (Cat. No. FBS-BBT, Rocky Mountain Biologicals, Missoula, MT, USA); 1% penicillin-streptomycin solution (Cat. No. PSL01-100ML, Caisson Labs, Smithfield, UT, USA); ITS Liquid Media Supplement (100X) (Cat No. I3146-5ML, Sigma-Aldrich, St. Louis, MO, USA) and Dexamethasone (Cat. No. D1756, Sigma-Aldrich, St. Louis, MO, USA). The cells were grown and maintained in T-75 culture flasks and incubated at 37 °C in 5% CO_2_. All experiments were performed in 6-well plates.

#### 2.1.2. Hep3B Hepatocytes

Human hepatocellular carcinoma Hep3B (liver epithelial) cells were purchased from American Type Culture Collection (ATCC) (Cat. No. HB-8064, ATCC, Manassas, VA, USA). The cells were grown in EMEM media (Cat. No. 30-2003, ATCC, Manassas, VA, USA) supplemented with 10% Fetal Bovine Serum (FBS) (Cat. No. FBS-BBT, Rocky Mountain Biologicals, Missoula, MT, USA) and 1% penicillin-streptomycin solution (Cat. No. PSL01-100ML, Caisson Labs, Smithfield, UT, USA). The cells were grown and maintained in T-75 culture flasks, incubated at 37 °C with 5% CO_2_. All experiments were performed in 6-well plates.

### 2.2. MC-LR Exposure & Antioxidant Treatment

The AML-12 hepatocytes were allowed to reach the desired confluency, after which they were serum-starved for 3 h at 37 °C with 5% CO_2_. Short-term serum starvation was done to synchronize the cell cycles to the quiescent phase and reduce background interference from serum components before exposure to the toxin. For exposure to MC-LR, lyophilized MC-LR (Cat. No. 10007188, Cayman Chemicals, Ann Arbor, MI, USA) was dissolved in 1 mL of Ultrapure Molecular-Grade water to obtain a final stock concentration of 1 mg/mL. Cells were treated with a final concentration of 10 µM MC-LR. To see the effect of antioxidants on MC-LR-induced hepatotoxicity, two antioxidants were selected—pNaKtide (custom-made by Ohio Peptide LLC, Powell, OH, USA), an in-house developed peptide [39,40,41], and N-acetylcysteine (NAC) (Cat. No. 616-91-1, Sigma-Aldrich, St. Louis, MO, USA), a clinically used drug. pNaKtide was used at 1 µM, whereas NAC was used at a final concentration of 10 mM for cell treatment, and both antioxidants were added 30 min prior to the addition of MC-LR. The control wells received an equal volume of 1X Phosphate-Buffered Saline (PBS) (Cat. No. ICN1860454, Fisher Scientific, Waltham, MA, USA). After the addition of reagents, the cells were incubated at 37 °C with 5% CO_2_ for 24 h before proceeding with further experimental analysis. An *n* = 3 was used for all groups.

### 2.3. MC-LR Exposure, MASLD Model & Antioxidant Treatment

The MASLD model was developed using Hep3B hepatocytes. After the cells reached the desired confluency, they were serum-starved for 3 h at 37 °C with 5% CO_2_ to synchronize the cell cycles and reduce serum-induced background interference. Oleic acid (OA) (Cat. No. O1383-1G, Sigma-Aldrich, St. Louis, MO, USA) was used to induce and mimic MASLD-like conditions in these cells [26]. A working stock solution of OA was prepared by dissolving the OA in 200 Proof Absolute Ethanol, Molecular Biology Grade (Cat. No. BP28184, Fisher Scientific, Waltham, MA, USA) to obtain a concentration of 50 mM. MASLD-like conditions were induced by adding a final concentration of 500 µM OA in each well, and the plates were incubated at 37 °C with 5% CO_2_ for an additional 24 h. An equivalent Ethanol control without OA was also set up. The MASLD phenomenon was evaluated for lipid droplet accumulation using Oil Red O staining, as described in the next section. To observe the adverse effects of cyanotoxin, a final concentration of 10 µM MC-LR was used for exposure. To determine the effect of antioxidants on MC-LR-induced hepatotoxicity under MASLD conditions, two antioxidants were used—pNaKtide and NAC. Concentrations of both antioxidants were the same as stated above. The MC-LR and antioxidant treatments were performed as described earlier, 24 h after OA treatment. After reagent addition, the cells were incubated at 37 °C with 5% CO_2_ for an additional 24 h before proceeding with further experimental analysis. An n = 3 was used for all groups.

### 2.4. Assessment of the Establishment of the MASLD Model In Vitro

#### 2.4.1. Oil Red O (ORO) Staining

ORO staining was performed according to the standard protocol. An ORO stock solution was prepared by dissolving 0.5 g of ORO powder (Cat. No. O0625, Sigma-Aldrich, St. Louis, MO, USA) into 100 mL of isopropanol (Cat. No. A426F-1GAL, Fisher Scientific, Waltham, MA, USA). A working solution was freshly prepared by adding 6 mL of stock to 4 mL of double-distilled water, and the mixture was filtered using Whatman paper. After treatment, the spent media was removed, and the cells were gently rinsed with 1X PBS. The cells were then fixed in 10% formalin for 30 min. Following fixation step, formalin was discarded, and the cells were washed twice with distilled water. Next, the cells were treated with 60% isopropanol for 5 min, followed by staining with the ORO solution for 10–20 min. The ORO stain was discarded, and the excess stain was washed off with distilled water. The cells were then incubated with the Modified Harris hematoxylin staining solution (Cat. No. 3530-16, Fisher Scientific, Waltham, MA, USA) for 1 min. Excess hematoxylin dye was washed off with distilled water, and the cells were examined for fat accumulation. Images of the ORO-stained cells were captured at 20x using an Olympus CKX53 microscope with Olympus cellSens Standard 1.15 (2016, version 1.15, Center Valley, PA, USA).

#### 2.4.2. Free Fatty Acid Assay

Free fatty acid (FFA) levels were quantified in Hep3B hepatocytes using the commercially available Free Fatty Acid Assay Kit (Cat No. ab65341, Abcam, Cambridge, UK). Sample preparation and assay were performed as per the manufacturer’s instructions.

#### 2.4.3. Triglyceride Assay

Triglyceride concentrations were quantified in Hep3B cells using the commercially available Triglyceride Quantification Assay Kit (Cat No. ab65336, Abcam, Cambridge, UK). Sample preparation and assay were performed as per the manufacturer’s instructions.

### 2.5. Assessment of Liver Injury

#### Human PAI1 (SERPINE1) ELISA

SERPINE1 was measured in the spent media of Hep3B hepatocytes using the commercially available Human PAI1 SimpleStep ELISA^®^ Kit (SERPINE1) (Cat No. ab269373, Abcam, Cambridge, UK) as per the manufacturer’s protocol.

### 2.6. Assessment of Oxidative Stress

#### 8-Hydroxy-2-Deoxyguanosine (8-OHdG) Assay

8-OHdG levels, a marker of oxidative stress and DNA damage, were assessed in the spent media of Hep3B hepatocytes using the commercially available 8-hydroxy-2′-deoxyguanosine (8-OHdG) ELISA Kit (Cat. No. ab285254, Abcam, Cambridge, UK) as per the manufacturer’s instructions.

### 2.7. RNA Extraction and Real-Time PCR Analysis

RNA extraction from the cells was performed using RNeasy Plus Mini Kit (Cat. No. 74136, Qiagen Sciences Inc., Germantown, MD, USA). Approximately 500 ng RNA was used to synthesize cDNA using QIAGEN’s RT^2^ First Strand Kit (Cat. No. 330401, Qiagen, Sciences Inc., Germantown, MD, USA). An automated, high-precision liquid handling workflow system, QIAgility, was used for qPCR sample and reagent loading. Qiagen Rotor-Gene Q thermo-cycler was used to run qPCR cycles. 18S rRNA (Cat. No. 4319413E, Thermo Fisher Scientific, Waltham, MA, USA) was used as a housekeeping gene for normalization. The following Taqman primers obtained from Thermo Fisher Scientific, Waltham, MA, USA were used to assess hepatotoxicity in AML-12 cells: *SerpinE* (Mm00435858_m1), *OSMR* (Mm01307326_m1), and Glutathione-S-transferase (*GST*) isoforms: *Gstm5* (Mm00515890_m1), *Gstm2*, and *Gstp1* (custom-made SYBR Green primers). As for the Hep3B cells, the following Taqman primers were used to assess inflammation and hepatotoxicity: *TNFα* (Hs174128_m1), *Tgfβ1* (Hs00998133_m1), *CD36* (Hs00354519_m1), *ALPL* (Hs01029144_m1), *OSMR* (Hs00384276_m1), and *SERPINE*1 (Hs00167155_m1). All qPCR experiments were performed in duplicate.

### 2.8. Mass Spectrometric Analysis

All solvents, namely, acetic acid, acetonitrile, formic acid, methanol, and water, were of Optima LC–MS grade and were purchased from Fisher Scientific (Tewksbury, MA, USA). MC-LR was purchased from Enzo Life Sciences, Inc. (Farmingdale, NY, USA). The surrogate C2D5 MC-LR was purchased from Cambridge Isotope Laboratories, Inc. (Tewksbury, MA, USA). The Westrick Group synthesized Microcystin LR-Cysteine [42]. Concentration of MC-LR-cysteine was determined by dissolving MC-LR cysteine into methanol and using the MC-LR absorptivity coefficient 39,800 L/cm mole at 238 nm.

The cells and supernatant samples were stored at −80 °C. Cell pellets were resuspended in PBS and rinsed three times with 1 mL of PBS. After the supernatant was removed, 0.5 mL of water was added, and the samples were lyophilized. Subsequently, 1.5 mL of water was added, vortexed, and centrifuged at 1000× *g* for 10 min. The supernatant was transferred to a 2 mL LC vial for analysis.

Online concentration LC-MS/MS was used to determine the concentrations of MC-LR and MC-LR Cysteine as described in Birbeck et al. [43]. Briefly, using a Thermo Scientific TSQ Altis™ 245 triple quadrupole mass spectrometer (Thermo Scientific, Waltham, MA, USA) with an EQuan 246 MAX Plus™ system, 1 mL of sample was injected onto a loading column (Thermo Scientific 247 Hypersil GOLD aQ 2.1 × 20 mm, 12 μm particle size) using an HTC PAL autosampler (CTC 248 Analytics, Zwingen, Switzerland). The analytical column used was a Thermo Accucore aQ (50 × 249 2.1 mm, 2.6 μm particle size column) and was maintained at a stable temperature of 35 °C for gradient analysis. Mass spectrometry analysis was performed using a positive electrospray ionization (ESI) source in 251 mode. Quantitation data results were obtained using TraceFinder™ EFS 4.1.

### 2.9. Statistical Analysis

Statistical analysis was done using GraphPad PRISM 7 (version 7.0, San Diego, CA, USA). Comparison within different experimental groups was done using either the 2-tailed Unpaired Student’s *t*-Test (for comparison between 2 groups) or the Ordinary One-way Analysis of Variance (ANOVA), specifically Tukey’s multiple comparison test (for three or more groups). All data are presented as Mean ± Standard Error of Mean (S.E.M.), and a *p* value of <0.05 was considered to be statistically significant.

## 3. Results

### 3.1. MC-LR Causes Hepatotoxic Effects in Mouse Liver Cells

**Figure 1:**
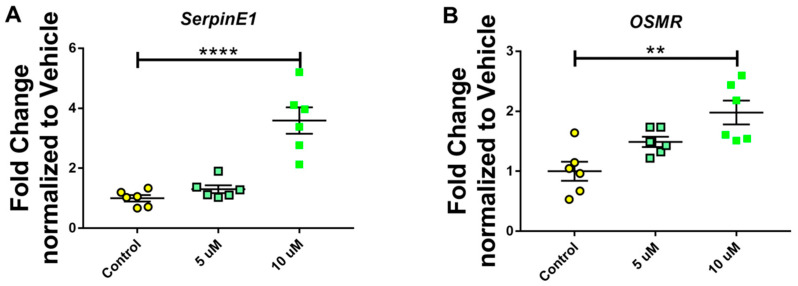
Effect of different doses of MC-LR on AML-12 hepatocytes. Exposure of AML-12 hepatocytes to 10 µM of MC-LR significantly increased the genetic expression of *SerpinE* (**A**) and *OSMR* (**B**), both of which are markers of hepatotoxicity. (*n* = 3, Duplicated; ** *p* ≤ 0.01, **** *p* ≤ 0.0001).

To determine the appropriate dose of MC-LR that is capable of inducing quantifiable hepatotoxicity, we exposed AML-12 hepatocytes to either 5 or 10 µM of MC-LR for 24 h. qPCR analysis for the markers of hepatotoxicity revealed that exposing the cells to 10 µM MC-LR significantly upregulated the expression of *SerpinE* and *OSMR*, both of which are markers of hepatotoxicity (Figure 1A,B).

### 3.2. Treatment with Antioxidants Reduces MC-LR-Induced Hepatotoxicity

**Figure 2:**
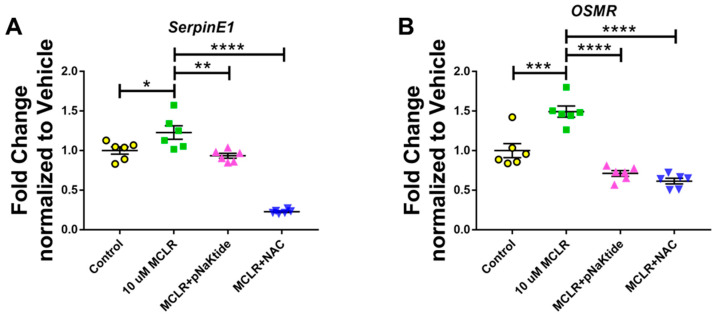
Targeted antioxidant treatment using pNaKtide and NAC significantly reduces MC-LR-induced hepatotoxicity. Exposure of AML-12 hepatocytes to 10 µM of MC-LR significantly increased the genetic expression of *SerpinE* (**A**) and *OSMR* (**B**), both of which are markers of hepatotoxicity, and were significantly downregulated on treatment with antioxidants—Src Kinase inhibitor pNaKtide and NAC (*n* = 3, Duplicated; * *p* ≤ 0.05, ** *p* ≤ 0.01, *** *p* ≤ 0.001, **** *p* ≤ 0.0001).

To determine if antioxidants—pNaKtide and NAC—can help reduce hepatotoxicity induced by MC-LR, we treated the cells with one µM pNaKtide and 10 mM NAC 30 min before exposing them to 10 µM of MC-LR for 24 h. qPCR analysis for the markers of hepatotoxicity revealed that exposing the cells to 10 µM MC-LR significantly upregulated the expression of *SerpinE* and *OSMR*, both of which were downregulated significantly on treatment with antioxidants (Figure 2A,B).

### 3.3. Genetic Analysis of GST Isoforms in Murine Hepatocytes

**Figure 3:**
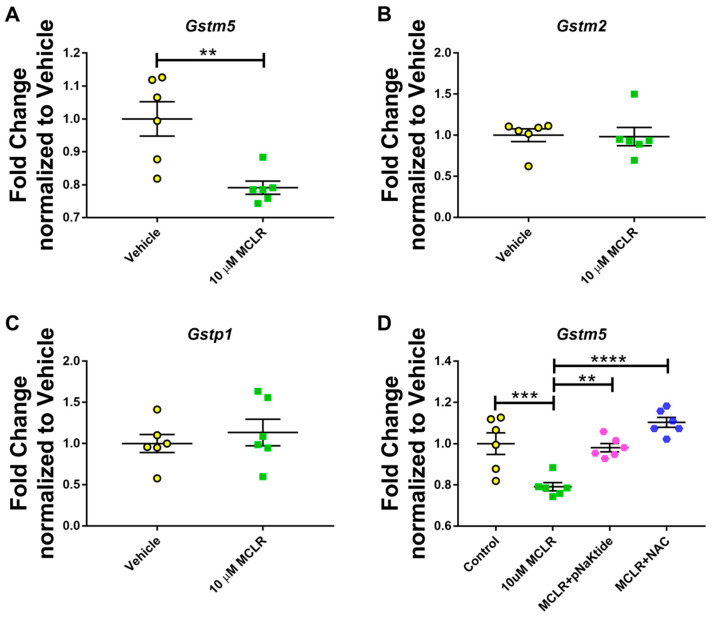
Quantitative PCR (qPCR) analysis of GST isoenzymes reveals the involvement of Gstm5 enzyme in response to MC-LR exposure. *Gstm5* (**A**) shows a significant downregulation on exposure to MC-LR; however, *Gstm2* (**B**), and *Gstp1* (**C**) remain unaffected in AML-12 hepatocytes. (**D**) Treatment with antioxidants significantly raised *Gstm5* expression after exposure to the toxin MC-LR. (*n* = 3, Duplicated; ** *p* ≤ 0.01, *** *p* ≤ 0.001, **** *p* ≤ 0.0001).

In our previous studies using animal models of MASLD, we observed that exposure to MC-LR induced additional stress, significantly lowering liver GST activity compared with the Control group [38]. Therefore, to determine how MC-LR affects the different isoforms of GST, we performed qPCR analysis for *Gstm2*, *Gstm5*, and *Gstp1*. Of the three isoforms tested, only *Gstm5* showed significant downregulation in the expression after exposure to MC-LR, whereas no changes were observed for either *Gstm2* or *Gstp1* (Figure 3A–C). Further analysis of *Gstm5* revealed that treatment of AML-12 hepatocytes with antioxidants after exposure to the toxin helped restore GST levels, as observed by a significant upregulation of Gstm5 expression in cells treated with pNaKtide and NAC compared to those exposed to the toxin (Figure 3D).

### 3.4. Oleic Acid Induces MASLD-like Conditions in Human Hepatocytes

**Figure 4:**
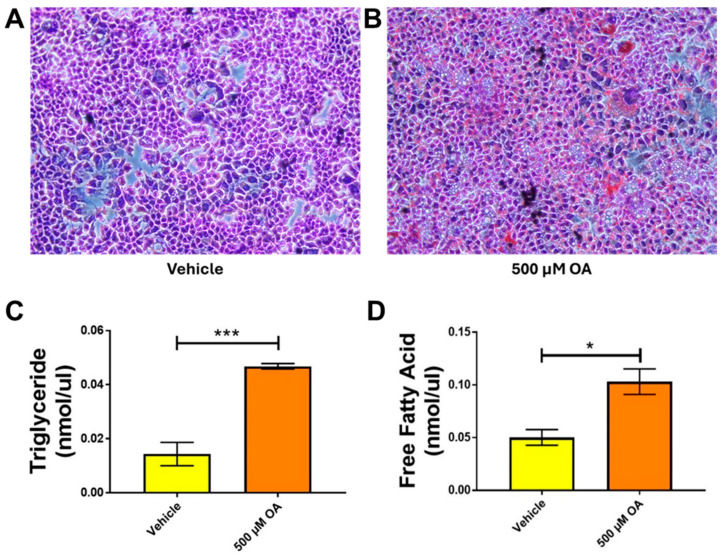
Treatment of Hep3B hepatocytes with 500 µM of oleic acid induces MASLD-like conditions. Representative pictures of ORO stained Hep3B cells (**A**,**B**) under 20x magnification shows accumulation of lipid droplets in cells treated with 500 μM of oleic acid, as well as significant increases in the levels of triglycerides (**C**) and free fatty acids (**D**) as compared to Vehicle (*n* = 3; * *p* ≤ 0.05, *** *p* ≤ 0.001).

To determine whether we can mimic MASLD in an in vitro setting and examine the role of hepatocytes, we treated Hep3B cells with 500 µM OA for 24 h. A simple ORO staining revealed noticeable lipid droplet accumulation in cells treated with oleic acid compared to the untreated Vehicle (Figure 4A,B; the original pictures are included in the Supplemental Figure S1A,B). It is well-known that MASLD increases triglyceride and free fatty acid levels in the body. Therefore, we quantified TG and FFA levels in OA-treated hepatocytes. Quantification of TG and FFA revealed a significant increase in OA-treated samples compared with Vehicle (Figure 4C,D).

### 3.5. Treatment with Antioxidants Ameliorates MC-LR-Induced Hepatotoxicity in MASLD Conditions

**Figure 5:**
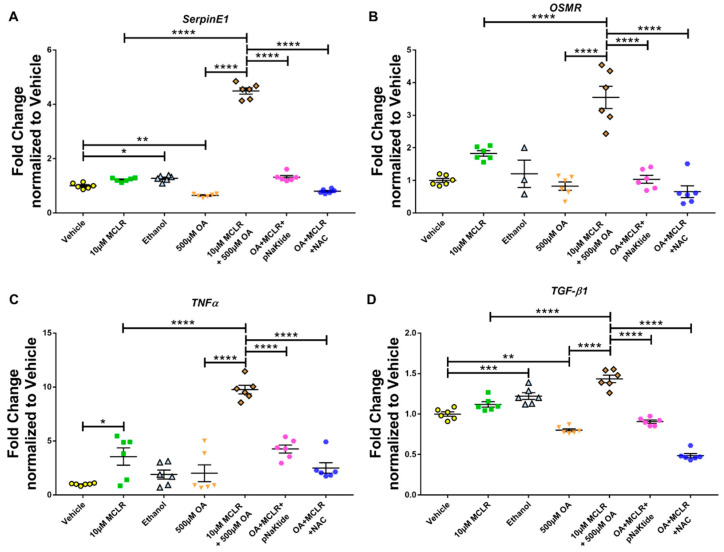

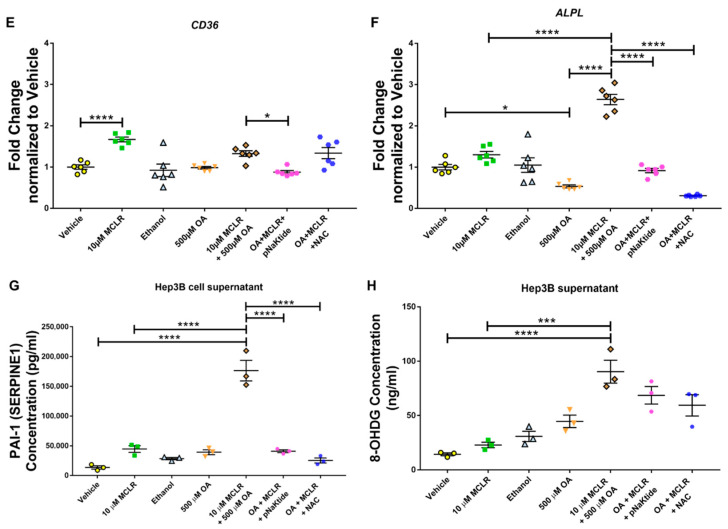
Targeted antioxidant treatment using pNaKtide and NAC significantly reduces MC-LR-induced hepatotoxicity in Hep3B hepatocytes. Quantitative PCR analysis of markers of hepatotoxicity—SerpinE1 (**A**) and OSMR (**B**); inflammation—TNFα (**C**) and Tgf-β1 (**D**); fatty acid metabolism—CD36 (**E**) and hepatic injury—ALPL (**F**) were all significantly upregulated on treatment with MC-LR (10 µM) in the presence of oleic acid (500 µM) as compared to those in the absence of oleic acid, except for CD36, which was upregulated in the presence of MC-LR alone. These markers were significantly downregulated in cells treated with targeted antioxidants. Quantification of SERPINE1/PAI-1 protein by ELISA (**G**) in the Hep3B supernatants mirrored the genetic expression of SerpinE1 and was also reduced with antioxidant treatment; similarly, 8-OHdG (**H**), a marker of oxidative stress, followed the same trend with MC-LR causing a significant oxidative stress in the presence of oleic acid as compared to MC-LR alone, and this was reduced with antioxidant treatment. (*n* = 3, with duplicates; * *p* ≤ 0.05, ** *p* ≤ 0.01, *** *p* ≤ 0.001, **** *p* ≤ 0.0001).

Our previous studies in animals with genetic or diet-induced MASLD exposed to MC-LR have shown a significant increase in the expression of markers of inflammation and hepatotoxicity. Therefore, we tested several markers and evaluated their expression under different conditions. As mentioned earlier, *SerpinE*1 and *OSMR* are markers of hepatotoxicity. Exposure of Hep3B hepatocytes to MC-LR alone upregulated the expression, although the increase was not significant. OA that was used to induce MASLD did not alter the expression of either *SerpinE*1 or *OSMR*, nor did Ethanol control. However, MC-LR in the presence of OA significantly upregulated the expression of both markers of hepatotoxicity, which was significantly downregulated on treatment with antioxidants (Figure 5A,B). We next assessed some markers of inflammation. Exposure to MC-LR significantly upregulated *TNFα* expression by 5-fold. Still, exposure to MC-LR in the presence of OA significantly upregulated the expression 10-fold higher than that of the Vehicle control. Treatment with antioxidants downregulated the expression of *TNFα* (Figure 5C) significantly. *Tgf-β1* showed a pattern similar to that of *SerpinE1* and *OSMR*: its expression was significantly upregulated upon toxin exposure in the presence of OA but significantly downregulated following antioxidant treatment (Figure 5D). *CD36*, which is associated with and directly contributes to the development of fatty liver, was significantly upregulated following exposure to the toxin alone. However, the expression was not significantly altered when cells were exposed to the toxin in the presence of OA (Figure 5E). We also assessed changes in *ALPL* gene expression, a clinical marker of liver tissue damage. *ALPL* expression was significantly upregulated when cells were exposed to the toxin in the presence of OA. These levels were significantly reduced to normal levels on treatment with antioxidants, pNaKtide, and NAC (Figure 5F). To corroborate the gene expression data, we quantified SERPINE1 (PAI-1) protein levels in the spent media from Hep3B hepatocytes (Figure 5G). We observed a significant increase in protein in the spent media from cells treated with both OA and MC-LR compared to cells treated with MC-LR alone. These levels were significantly reduced in cells treated with antioxidants. MC-LR is a known inducer of oxidative stress. Therefore, we quantitatively determined the free 8-OHdG levels, an oxidative derivative of guanosine and a stable marker of oxidative stress, in the spent media (Figure 5H). We observed a significant increase in 8-OHdG levels in the spent media of MASLD cells exposed to the toxin compared with those exposed to MC-LR alone. Treatment with targeted antioxidants showed a decrease in the 8-OHdG levels as compared to OA + MC-LR exposed group. These findings indicate the role of targeted antioxidants in mitigating MC-LR-induced hepatocyte toxicity.

### 3.6. Targeted Antioxidant Treatment Aids in MC-LR Metabolism by the Hepatocytes

**Figure 6:**
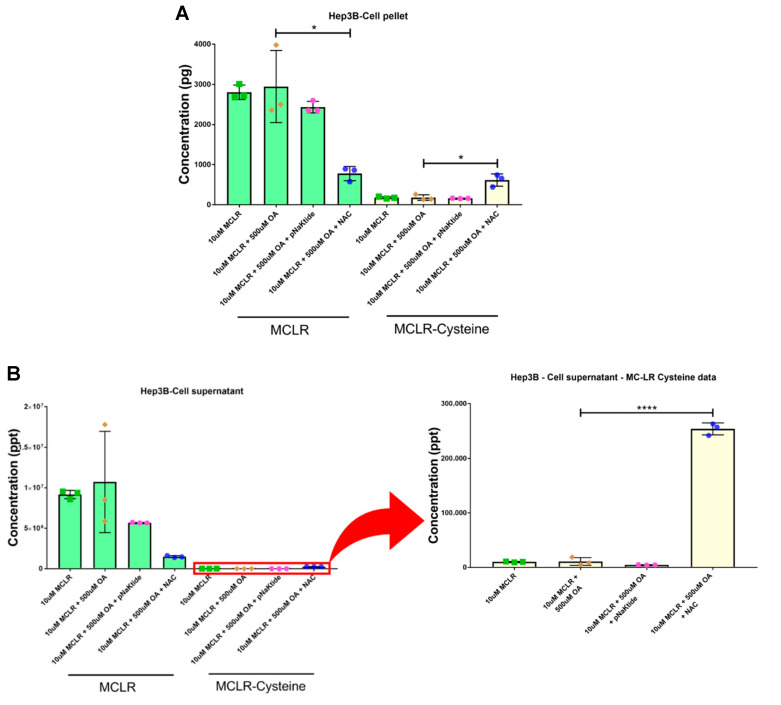
Targeted antioxidant treatment significantly improves MC-LR metabolism in hepatocytes. Mass spectrometric-based quantification of MC-LR (green bars) and MC-LR Cysteine (yellow bars) in Hep3B cell pellet (**A**) and Hep3B cell supernatant (**B**) showed a decrease in the levels of MC-LR in cells that were treated with antioxidants as compared to the MASLD cells exposed to the toxin. On the other hand, the levels of detoxified MC-LR Cysteine were improved on treatment with antioxidants (inset to right shows MC-LR-Cysteine data on a separate scale). (*n* = 3; * *p* ≤ 0.05, **** *p* ≤ 0.0001).

To determine the role of hepatocytes in toxin metabolism, we developed a mass spectrometry-based method to measure the concentrations of MC-LR and its detoxified metabolite, MC-LR-cysteine, in cell pellet homogenates and spent media. Our previous in vivo studies showed that chronic low-dose exposure to MC-LR in the MASLD setting affected the breakdown of MC-LR to its detoxified version, MC-LR-Cysteine, via the glutathione pathway [37]. Both pNaKtide and NAC helped in improving the glutathione-S-transferase (GST) levels, in turn helping with the GST-mediated breakdown of MC-LR to MC-LR Cysteine [38]. Therefore, we quantified the levels of complete MC-LR and its detoxified metabolite, MC-LR Cysteine, in Hep3B cell pellets (Figure 6A) and spent media (Figure 6B). Although pNaKtide did not show any significant changes in metabolism, cells treated with NAC were better able to metabolize MC-LR than MASLD-induced cells exposed to the toxin. This suggests that antioxidant therapy can support hepatocytes in metabolizing toxins.

## 4. Discussion

In our previous study, we observed that short-term exposure to low-dose of MC-LR in diet-induced MASLD mice significantly increased inflammation, immune cell infiltration, oxidative stress as well as affected the toxin metabolism and the genetic expression of the markers of drug transporters and Phase I and II metabolizing enzymes in the liver and that treatment with antioxidants such as the specific Src-kinase inhibitor pNaKtide and NAC played an essential role in alleviating these effects. Since the liver is composed of several different cell types that are involved in various functions, such as metabolism and immune response, we aimed to specifically investigate the role of hepatocytes in the detoxification of toxins, particularly in the context of MASLD. In this study, we used two established cell lines derived from murine and human hepatocytes. Cells exposed to MC-LR, with or without the antioxidants—pNaKtide and NAC, exhibited a significant increase in hepatotoxicity markers following toxin exposure, but were downregulated with antioxidants. To study steatosis in hepatocytes, the cells were first treated with OA before exposure to toxins or antioxidants. MC-LR exposure in MASLD settings significantly increased expression of hepatotoxicity and inflammation markers, compared with exposure to the toxin in the absence of MASLD, and this effect was significantly attenuated by antioxidant treatment.

A wide variety of in vitro models are being developed to understand better and assess the risk of lipotoxicity and disease progression [44]. Although primary hepatocytes are generally considered among the best in vitro models, donor variability and cell alterations after isolation are significant sources of variability in experimental results. Therefore, for this study, we have used two different hepatocyte cell lines—AML-12 cells, a murine liver cell line, and Hep3B, a human hepatocellular carcinoma cell line. Since our in vivo studies used a murine model. The AML-12 cell line originated from normal mouse liver epithelial cells, and is a suitable model for studying the role of hepatocytes in MC-LR-induced hepatotoxicity. To assess the relevance of the effects of MC-LR-induced hepatotoxicity in human liver, we used the Hep3B cell line derived from human hepatocellular carcinoma. Both these cell lines are well-established models used to study drug toxicity and various mechanistic studies.

Preliminary studies conducted in AML-12 hepatocytes to determine the dose of MC-LR revealed that a final concentration of 10 µM could induce significant toxicity in the hepatocytes without affecting their viability. This was evaluated by genetic analysis of markers of hepatotoxicity—*SerpinE* and *OSMR*, identified in our previous study [37]. *SerpinE1* is a serine protease inhibitor, a principal inhibitor of tissue plasminogen activator (tPA), and is usually involved in the immune response [45]. *OSMR*, on the other hand, is a cytokine-responsive gene often associated with inflammation and apoptosis [46]. To determine if MC-LR-induced hepatotoxicity could be ameliorated with antioxidants, as observed in our in vivo studies [38], we treated the cells with pNaKtide or NAC prior to exposure to MC-LR. pNaKtide is an in-house developed peptide sequence that binds to the kinase domain of sarcoma-related kinase (Src), thus inhibiting its function [47,48]. Activation of the Src protein is associated with numerous disease states and an increase in reactive oxygen species [49]. Kutz et al. demonstrated that pharmacological inhibition of the Na^+^/K^+^-ATPase α1/Src interaction with pNaKtide enhanced metabolic reserve and mitigated Western diet-induced intolerance in vivo, consistent with the suppression of downstream oxidant signaling [39,40,41]. NAC is an acetylated variant of L-cysteine and is a precursor of cellular reduced glutathione (GSH). It helps in stimulating GSH biosynthesis and is widely used clinically for the treatment of hepatotoxicity in cases of GSH deficiency [50,51,52]. Our study demonstrated that treating AML-12 hepatocytes with antioxidants after exposure to the cyanotoxin MC-LR significantly reduced MC-LR-induced hepatotoxicity.

It is well-established that GST activity is usually upregulated during oxidative stress [53]. In our previous studies using a murine model of diet-induced MASLD, we observed that exposure to MC-LR significantly reduced glutathione S-transferase (GST) activity, which was restored upon antioxidant treatment. Gehringer et al. conducted a study to investigate the hepatotoxic effects of MC-LR on lipid peroxidation and glutathione detoxification at the transcriptional and protein activity levels. It was reported that glutathione peroxidase activity was significantly increased at 24 and 32 h post-exposure to MC-LR, as well as the gene expression of GST isoenzymes—*Gstm2*, *Gstm5*, and *Gstp1* were reported to be upregulated 8 h post-exposure [54]. However, in our study, we observed that exposure to MC-LR for 24 h significantly downregulated the genetic expression of *Gstm5*, but no alterations in the expression of *Gstm2* and *Gstp1*. Interestingly, *Gstm5* expression was significantly upregulated and restored in cells exposed to MC-LR and treated with pNaKtide or NAC. These results align with our observations of GST activity in diet-induced MASLD mice exposed to the toxin and/or treated with antioxidants, where GST activity was significantly decreased upon exposure to the toxin but were restored upon treatment with antioxidants.

MASLD is one of the most common liver conditions in Western countries, affecting ≥30% of the population. To study this phenomenon and assess the role played by hepatocytes, we treated Hep3B hepatocytes with Oleic acid, the most abundant dietary and circulatory fatty acid, which is commonly used to induce the MASLD phenotype in in vitro models [55,56]. A study done by Araya et al. noted that patients with hepatic steatosis presented accumulation of excess oleic acid, an end product of de novo fatty acid synthesis [57]. In our studies, we discovered that experiments studying the effect of OA-induced steatosis and MC-LR toxicity produced the most significant results in Hep3B cells. Quantification of triglycerides and free fatty acids in Hep3B cells treated with OA revealed substantial increases in levels compared to the untreated Control group. Various studies have shown that the induction of MASLD using OA in vitro increases the levels of triglycerides and free fatty acids [58,59]. We have previously shown that mice with pre-existing MASLD are more susceptible to MC-LR-induced hepatotoxicity, which can be reduced on treatment with antioxidants such as pNaKtide and NAC. Therefore, we aimed to investigate this in an in vitro model to verify whether hepatocytes also contribute to the reduction in MC-LR-related hepatotoxicity. We assessed the genetic expression of various markers associated with hepatotoxicity and inflammation. To assess hepatotoxicity, we evaluated *SerpinE*1 and *OSMR* and found that both genes were significantly upregulated on exposure to MC-LR in the presence of OA-induced MASLD. Interestingly, this upregulation was several times higher in the presence of OA and MC-LR as compared to cells that were only exposed to the toxin. These levels were significantly downregulated on treatment with antioxidants. We also performed an ELISA to quantify PAI1 (SERPINE1) protein levels in the spent media of Hep3B hepatocytes and observed the same pattern as the genetic expression of *SerpinE1*. MC-LR is known to modulate immune responses and inflammatory cytokine expression in several animal models, such as mice and fish [18]. A study done by Yoshida et al. in mice that were given a single intraperitoneal injection of 60 µg/kg of MC-LR and euthanized at various time points showed that the genetic expression of *TNFα* was 2.3-fold higher than that of the controls [60]. This agrees with our observation of the *TNFα* levels in our study with Hep3B cells, wherein exposure to MC-LR increased the level ~3 fold, and exposure to the toxin with OA increased the levels ~10 fold as compared to the untreated control. These levels were significantly attenuated following treatment with antioxidants. Another standard marker associated with MC-LR toxicity is *Tgf-β1*, which is more associated with liver fibrosis. A study done by Arman et al. in mice showed that the presence of steatosis alone significantly upregulates the expression of *Tgf-β1*, but the addition of MC-LR did not alter the expression significantly [35]. On the contrary, our results showed that exposure of Hep3B cells to MC-LR increased the levels of *Tgf-β1*. Induction of steatosis with OA did not alter the levels of the gene in any way, but significantly increased the expression when exposed to the toxin in steatosis conditions. Both pNaKtide and NAC were able to significantly downregulate the gene expression as compared to the MC-LR-exposed cells. *CD36* plays an important role in the uptake of fatty acids, and deletion of this gene has been shown to induce steatosis in animals [61,62,63]. In their animal studies, Arman et al. have shown that exposure to 30 µg/kg of MC-LR in animals fed a high-fat diet significantly increased *CD36* levels [35]. In our in vitro study, we observed that exposure to MC-LR alone significantly increased *CD36* expression, and we observed a trend toward increased *CD36* expression in MASLD cells exposed to the toxin. Another gene associated with hepatic injury is alkaline phosphatase. This is a clinically used marker to assess liver damage, and an increase in the expression of this gene is associated with hepatotoxicity. We have previously shown that treatment of MASLD mice exposed to different doses of MC-LR showed a dose-dependent increase in alkaline phosphatase levels [37]. In agreement with this, our in vitro studies showed significant upregulation of the alkaline phosphatase gene in OA-treated cells that were exposed to the toxin. These levels were significantly downregulated on treatment with antioxidants. MC-LR is a well-known inducer of oxidative stress. To quantify this response and determine whether the antioxidants—pNaKtide and NAC reduce MC-LR-induced oxidative stress, we performed an 8-OHdG assay using spent media from Hep3B hepatocytes. 8-OHdG is an oxidative derivative of guanosine and is produced upon oxidative damage of DNA by reactive oxygen and/or nitrogen species. This process arises from both normal metabolic activity and various environmental factors. Increased 8-OHdG levels have been linked to several pathological conditions, including atherosclerosis, cancer, diabetes, hypertension, and steatosis [64]. In our study, we observed that the levels of 8-OHdG were significantly higher in MASLD cells that were exposed to the toxin as compared to non-MASLD MC-LR-exposed cells. These levels, though not significant, were reduced in groups that were treated with antioxidants. These findings suggest that antioxidants might be useful in ameliorating MC-LR’s toxicity in this setting. We also developed a mass spectrometric approach to detect and quantify the levels of the toxin and its metabolite, MC-LR Cysteine, in the spent media and cell pellets, and the results are in accordance with the GST activity, i.e., MC-LR levels were seen to be significantly decreased on treatment with antioxidants, whereas there was a trend if increasing levels of the MC-LR Cysteine metabolite. This indicates that MASLD, in combination with MC-LR, may reduce glutathione and related enzyme levels, thereby affecting the metabolism of the toxin, a condition restored by antioxidant treatment.

## 5. Conclusions

In summary, hepatocytes play a critical role in the metabolism of xenobiotic compounds such as MC-LR, and pre-existing conditions such as MASLD significantly increase hepatocyte susceptibility to cyanotoxins like MC-LR. In our study, we demonstrated that exposure to MC-LR significantly increased hepatotoxicity as evidenced by the upregulation of *SerpinE* and *OSMR* in both AML-12 and Hep3B cells. Since the presence of both MASLD and MC-LR is known to contribute to oxidative stress, we studied the genetic expression of GST isoenzymes and found that only *Gstm5* was downregulated upon exposure to MC-LR, whereas *Gstm2* and *Gstp1* expression levels remained unchanged. Further, treatment with antioxidants, pNaKtide, and NAC restored Gstm5 expression. We successfully mimicked a MASLD-like phenomenon in Hep3B cells using OA, as confirmed by increased lipid accumulation detected by ORO staining. Exposure to MC-LR in the presence of MASLD setting significantly upregulated markers of hepatotoxicity (*SerpinE*1 and *OSMR*), inflammation (*TNFα* and *TGF-β1*), and liver injury (*ALPL*) compared with MC-LR exposure under normal conditions, indicating increased susceptibility to the cyanotoxin. We demonstrated that antioxidant treatment effectively reversed these changes. Mass spectrometric analysis of both cell pellet and cell supernatants showed a decreasing trend in the concentration of MC-LR toxin in groups treated with antioxidants compared to hepatocytes treated with MC-LR alone, along with an increasing concentration of the MC-LR Cysteine metabolite. These results highlight the critical role of hepatocytes in the metabolism of xenobiotic compounds such as MC-LR, and that treatment with targeted antioxidants, such as pNaKtide and NAC, could be developed as a potential therapeutic strategy to mitigate the adverse effects of MC-LR in the setting of pre-existing conditions like MASLD.

## 6. Limitations

This study employed immortalized cell lines: a mouse-derived hepatocyte line (AML-12) and a human-derived hepatocarcinoma cell line (Hep3B). Prior research relied on murine models of MASLD; however, establishing a functional MASLD phenotype in AML-12 cells proved unfeasible. Therefore, we utilized Hep3B cells, a well-established and widely accepted human cellular model of MASLD induced by oleic acid supplementation. Hep3B cells are suitable for investigating human-relevant hepatocyte responses to MC-LR because they retain key metabolic pathways and express many of the same transporters and detoxification enzymes found in human liver tissue. However, as an immortalized cell line, Hep3B cells also exhibit a dampened cellular response compared to primary human hepatocytes, including reduced sensitivity to oxidative stress and toxin-induced injury. As a result, higher MC-LR dosages were required in this model than would be typical of real-world environmental exposures.

Furthermore, mechanistic investigations into mitochondrial dysfunction, apoptosis, and the influence of interacting cell types (such as immune cells) were not conducted. Despite these limitations, this study is the first to demonstrate that hepatocytes under metabolic stress (MASLD) exhibit a heightened response to MC-LR and that targeted antioxidant therapies may represent promising therapeutic strategies.

## Data Availability

The original contributions presented in this study are included in the article/Supplementary Material. Further inquiries can be directed to the corresponding authors.

## Supplementary Materials

The following supporting information can be downloaded at https://www.mdpi.com/article/10.3390/jox16030076/s1: Figure S1: Original images of ORO-stained Hep3B cells.
