# Supplementary material for: Antioxidant Therapy Reverses Hepatotoxicity Induced by Microcystin-LR in a Cellular Model of Metabolic Dysfunction-Associated Steatotic Liver Disease (MASLD)
Source: J Xenobiot. 2026 Apr 29;16(3):76. doi: 10.3390/jox16030076 (PMC13214806; doi:10.3390/jox16030076)
Supplement: Supplementary file 1 [file jox-16-00076-s001.zip › jox-4169186-supplementary.pdf]

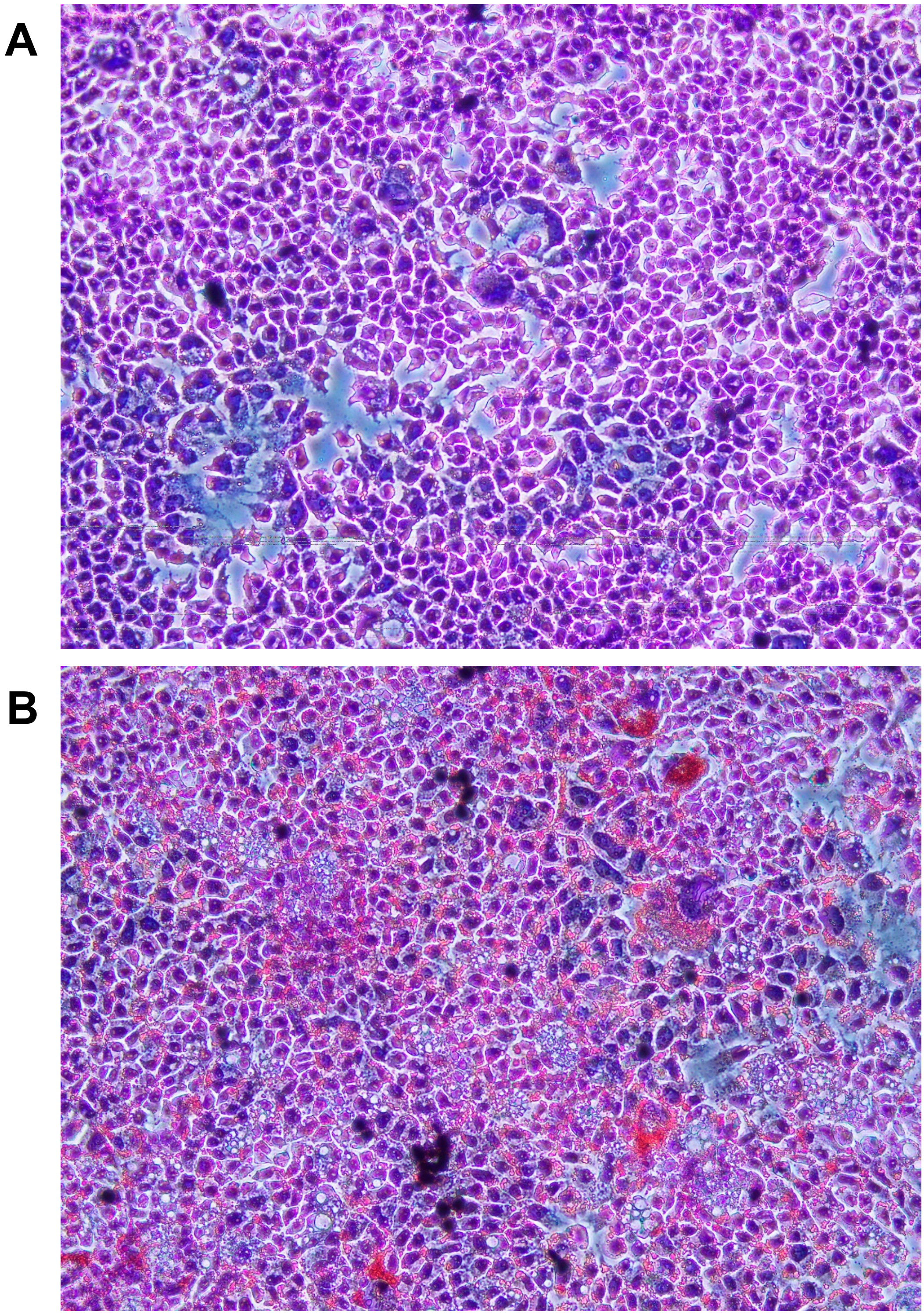

Supplementary Figure S1: Original images of ORO-stained Hep3B cells – Vehicle **(A)** and 500 µM Oleic acid (OA) treated **(B)** group showing increased accumulation of lipid droplets (in red) in OA treated hepatocytes. Images were captured at 20x using an Olympus CKX53 microscope and Olympus cellSens software.
